# Microbiome in Blood Samples From the General Population Recruited in the MARK-AGE Project: A Pilot Study

**DOI:** 10.3389/fmicb.2021.707515

**Published:** 2021-07-26

**Authors:** Patrizia D’Aquila, Robertina Giacconi, Marco Malavolta, Francesco Piacenza, Alexander Bürkle, María Moreno Villanueva, Martijn E. T. Dollé, Eugène Jansen, Tilman Grune, Efstathios S. Gonos, Claudio Franceschi, Miriam Capri, Beatrix Grubeck-Loebenstein, Ewa Sikora, Olivier Toussaint, Florence Debacq-Chainiaux, Antti Hervonen, Mikko Hurme, P. Eline Slagboom, Christiane Schön, Jürgen Bernhardt, Nicolle Breusing, Giuseppe Passarino, Mauro Provinciali, Dina Bellizzi

**Affiliations:** ^1^Department of Biology, Ecology and Earth Sciences (DIBEST), University of Calabria, Rende, Italy; ^2^Advanced Technology Center for Aging Research, IRCCS (Scientific Institute for Research, Hospitalization and Healthcare) INRCA National Institute on Health and Science on Ageing, Ancona, Italy; ^3^Molecular Toxicology Group, Department of Biology, University of Konstanz, Konstanz, Germany; ^4^Department of Sport Science, Human Performance Research Centre, University of Konstanz, Konstanz, Germany; ^5^Centre for Health Protection, National Institute for Public Health and the Environment, Bilthoven, Netherlands; ^6^Department of Molecular Toxicology, German Institute of Human Nutrition Potsdam-Rehbruecke (DIfE), Nuthetal, Germany; ^7^NutriAct-Competence Cluster Nutrition Research Berlin-Potsdam, Nuthetal, Germany; ^8^National Hellenic Research Foundation, Institute of Biology, Medicinal Chemistry and Biotechnology, Athens, Greece; ^9^Department of Experimental, Diagnostic and Specialty Medicine, Alma Mater Studiorum, University of Bologna, Bologna, Italy; ^10^Institute of Information Technologies, Mathematics and Mechanics, Lobachevsky University, Nizhny Novgorod, Russia; ^11^Interdepartmental Center, Alma Mater Research Institute on Global Challenges and Climate Change, University of Bologna, Bologna, Italy; ^12^Research Institute for Biomedical Aging Research, University of Innsbruck, Innsbruck, Austria; ^13^Laboratory of the Molecular Bases of Ageing, Nencki Institute of Experimental Biology, Polish Academy of Sciences, Warsaw, Poland; ^14^Research Unit of Cellular Biology (URBC) Namur Research Institute for Life Sciences (Narilis), University of Namur, Namur, Belgium; ^15^Medical School, University of Tampere, Tampere, Finland; ^16^Department of Molecular Epidemiology, Leiden University Medical Centre, Leiden, Netherlands; ^17^BioTeSys GmbH, Schelztorstr, Esslingen, Germany; ^18^Department of Applied Nutritional Science/Dietetics, Institute of Nutritional Medicine, University of Hohenheim, Stuttgart, Germany

**Keywords:** blood microbiome, 16S rRNA gene, geographic origin, aging, free fatty acids, leukocytes, insulin, glucose

## Abstract

The presence of circulating microbiome in blood has been reported in both physiological and pathological conditions, although its origins, identities and function remain to be elucidated. This study aimed to investigate the presence of blood microbiome by quantitative real-time PCRs targeting the 16S rRNA gene. To our knowledge, this is the first study in which the circulating microbiome has been analyzed in such a large sample of individuals since the study was carried out on 1285 Randomly recruited Age-Stratified Individuals from the General population (RASIG). The samples came from several different European countries recruited within the EU Project MARK-AGE in which a series of clinical biochemical parameters were determined. The results obtained reveal an association between microbial DNA copy number and geographic origin. By contrast, no gender and age-related difference emerged, thus demonstrating the role of the environment in influencing the above levels independent of age and gender at least until the age of 75. In addition, a significant positive association was found with Free Fatty Acids (FFA) levels, leukocyte count, insulin, and glucose levels. Since these factors play an essential role in both health and disease conditions, their association with the extent of the blood microbiome leads us to consider the blood microbiome as a potential biomarker of human health.

## Introduction

The human body is considered an ecosystem, consisting of trillions of bacterial, fungal and viral taxa that inhabit the skin, gastrointestinal, respiratory and urogenital tracts.

For years, researchers have been interested in the characterization of gut microbiome, which appears taxonomically and functionally peculiar in each part of the gastrointestinal tract and displays high inter-individual heterogeneity according to age, gender, and environmental factors, including dietary regime and antibiotic use (Yatsunenko et al., 2012; Tidjani Alou et al., 2016; Rinninella et al., 2019; D’Aquila et al., 2020). More recently, some studies revealed the presence of a “dormant,” not-immediately cultivatable blood microbiome in both physiological and pathological conditions (Castillo et al., 2019). The first evidence date back to the late 1960s, with the observation of bacterial forms in erythrocytes of healthy individuals (Tedeschi et al., 1969). Afterward, the presence of bacterial DNA and bacteria-like structures was evaluated in blood specimens of healthy human subjects as well as various other species (McLaughlin et al., 2002; Sze et al., 2014; Jeon et al., 2017; Vientós-Plotts et al., 2017). More particularly, Nikkari et al. (2001) detected members of seven phylogenetic groups and five bacterial divisions or subdivisions in the blood specimen of healthy individuals, with the majority of the 16S rDNA sequences highly similar to that of *Pseudomonas fluorescens*. An independent work has subsequently identified a variety of bacteria, namely those belonging to the *Aquabacterium, Stenotrophomonas, Budvicia, Serratia, Bacillus*, and *Flavobacterium subgroups* (Moriyama et al., 2008). Besides, transmission electron and dark-field microscopy as well as flow cytometry assays revealed the existence of pleomorphic bacteria exhibiting limited growth and susceptibility to antibiotics in the serum of healthy human subjects (McLaughlin et al., 2002). Viable bacteria, including *Propionibacterium acnes*, *Staphylococcus epidermidis*, *Staphylococcus caprae*, *Micrococcus luteus*, and *Acinetobacter lwoffii* were isolated in 53% of the plasma and 35% of the RBC-suspension samples from blood donors, suggesting the inability of conventional tests employed by blood banks to detect bacteria in standard blood-pack units (Damgaard et al., 2015). Païssé et al. (2016) implemented the above-reported evidence by characterizing the distribution of taxa both in whole blood and in the different blood fractions. Data revealed that the bacterial DNA mostly localizes to the buffy coat fraction (about 94%) and little in the plasma (0.03%) and that *Proteobacteria* represents the most frequent phylum in each fraction. In addition, the different blood fractions appeared characterized by different taxa, with the *Sphingobacteria* highly represented in buffy coat and plasma and *Clostridia* found mostly in plasma and the red blood fraction. The high similarity of results across independent studies revealed the existence of a core blood microbiome, in which *Proteobacteria* predominate, and *Actinobacteria*, *Firmicutes*, and *Bacteroidetes* are present to a lesser extent (Amar et al., 2013; Lelouvier et al., 2016; Païssé et al., 2016; Olde Loohuis et al., 2018; Whittle et al., 2019).

Similarly, pathological conditions have been associated with blood microbiota dysbiosis and disease-specific changes in its composition, thus suggesting its potential role as a biomarker for diseases risk (Potgieter et al., 2015; Traykova et al., 2017; Li et al., 2018; Dutta et al., 2019).

A longitudinal study carried out by Amar et al. (2011) revealed that the 16S rDNA concentration in blood appeared to be a good predictor of the onset of diabetes and abdominal adiposity in a general population. More recent evidence reported that, although no significant difference in the relative abundance of blood microbiome between type 2 diabetes mellitus T2DM patients and not-T2DM controls at both phylum and class levels, the genera *Sediminibacterium* and *Bacteroides* are significantly associated with an increased and decreased risk of T2DM, respectively (Sato et al., 2014; Qiu et al., 2019; Anhê et al., 2020). An increased proportion of *Proteobacteria* phylum in blood was also demonstrated to predict the occurrence of cardiovascular events, meanwhile an increased *Actinobacteria: Proteobacteria* ratio is a distinctive feature of cardiovascular disease patients (Amar et al., 2013; Dinakaran et al., 2014). Lastly, the microbial reactivation of dormant non-replicating blood bacteria was demonstrated to increase the risk of developing Alzheimer disease in response to iron dysregulation, by shedding potent inflammagens such as lipopolysaccharide (LPS) and lipoteichoic acid (LTA) that promote systemic inflammation, fibrin amyloidogenesis, blood brain barrier permeability and affect the hematological system (Kell and Pretorius, 2018; Pretorius et al., 2018).

Whether it has been widely reported that the gut microbiome evolves during the lifespan, differs between populations, and responds to changing lifestyles, there is still no evidence of this about the blood microbiome (Yatsunenko et al., 2012; Wilmanski et al., 2021). To address these gaps, we carried out a preliminary investigation of the abundance of blood microbiome in 1285 Randomly recruited Age-Stratified Individuals from the General population (RASIG) from several different geographical regions of Europe recruited within the EU Project MARK-AGE (HEALTH-F4-2008–200880 MARK-AGE).

MARK-AGE is a Europe-wide cross-sectional population study aimed at the identification of biomarkers of aging in subjects within the age range 35–75 (Bürkle et al., 2015; Capri et al., 2015; Moreno-Villanueva et al., 2015a).

Considering the availability of several biochemical and clinical data from the MARK-AGE project, another purpose of the present study was to evaluate the association between the circulating bacterial DNA and several laboratories and clinical parameters.

## Materials and Methods

### Study Population and Blood Sample Collection

The present study included 1285 RASIG (Randomly recruited Age-Stratified Individuals from the General population) participants recruited in the MARK-AGE cross-sectional study from 2008 to 2012 (Bürkle et al., 2015). Subjects (503 males and 782 females) were in the age range of 35–75 years and came from several European countries: Germany, Belgium, Poland, Greece, Austria, Italy and Finland (Capri et al., 2015). RASIG, representing the “normal” aging, were included in the project if they met the following criteria:-inclusion criteria: randomly recruited age-stratified individuals from the general population (both sexes) aged 35–75 and able to give informed consent.

-exclusion criteria: (i). self-reported seropositivity for HIV, HBV (except seropositivity by vaccination) and HCV (HBV and HCV seropositivity assessed after blood collection). (ii). presence of actual cancer and current use of anti-cancer drugs or glucocorticoids; (iii). less than 50% of a lifetime spent in the country of residence. iv. inability to give informed consent or (vi) any acute illness (e.g., common cold) within 7 days preceding blood collection (Bürkle et al., 2015; Capri et al., 2015). Participants were asked to classify their self-rated health (SRH) using a standard 5-point scale with the responses excellent, very good, good, fair, or poor (Assari et al., 2016; Osibogun et al., 2018). In order to obtain a comparable group size, those answering excellent and very good and those answering fair and poor were grouped, respectively (Piacenza et al., 2021). Ethical approval was given by the ethics committee of each of the recruiting center. Written informed consent was obtained from all participants (Moreno-Villanueva et al., 2015a). Other details of the recruitment procedures and the collection of anthropometric, clinical and demographic data have been published elsewhere (Moreno-Villanueva et al., 2015a, b; Jansen et al., 2015). This study utilized biobanked samples stored at –80°C as prescribed by the standard operating procedures (Moreno-Villanueva et al., 2015a). In brief, all biological samples were processed within 2 h and 10 min following a master protocol elaborated for MARK-AGE purposes. Anticoagulated whole blood, obtained by phlebotomy after overnight fasting, was collected shipped from the various recruitment centers to the MARK-AGE Biobank located at the University of Hohenheim (Stuttgart, Germany). Whole blood, serum and EDTA/LiHep plasma aliquots were prepared from monovette blood tubes and stored at −80°C immediately after cryotubes were filled. From the biobank, coded samples were subsequently shipped to the Scientific and Technological Pole of INRCA of Ancona, Italy, on dry ice, where they were stored at –80°C until use. For each proband, sample collection time, sample preparation starting time and sample freezing time were documented by the recruiting staff. Abnormalities like erythrocyte contamination in any samples were also recorded. In the recruiter centers of the MARK-AGE project, the sampling was performed according to a very strict protocol (see Capri et al., 2015; Moreno-Villanueva et al., 2015a). As a result, the quality of the serum and plasma samples was very good (Jansen et al., 2015).

### Determination of Clinical Biochemical and Laboratory Parameters

Clinical, laboratory and biochemical parameters were measured as previously described (Hosaka et al., 1981; Bürkle et al., 2015; Moreno-Villanueva et al., 2015b; Jansen et al., 2015). In particular, glucose, insulin and Free Fatty Acid (FFA) were measured by the LX20 autoanalyzer. Glucose and insulin assays were obtained from Beckman–Coulter (Woerden, Netherlands), whereas FFA ones from Wako Diagnostics (Neuss, Germany). All the assays on the LX20 were enzymatic with a colorimetric endpoint. FFA and glucose were measured in serum samples, whereas insulin in Li/Hep EDTA plasma aliquots (Jansen et al., 2015).

### 16S rRNA Quantification by Real-Time qPCR

DNA was extracted from 300 μl of whole blood biobanked samples using QIAamp DNA Blood mini kit (Qiagen GmbH, Germany) according to the manufacturer’s instructions.

Highly sensitive and specific universal primers targeting the V3-V4 hypervariable region of the bacterial 16S rDNA were used in real-time qPCR reactions to quantify the 16S rRNA gene copy number in DNA samples (Nadkarni et al., 2002; Qian et al., 2018). The PCR mixture (20 μL) consisted of 20 ng of DNA, SensiFAST SYBR Hi-ROX Mix 1X (Bioline, London, United Kingdom) and 0.4 μM of the following primers: For 5′-TCCTACGGGAGGCAGCAGT-3′ and Rev 5′-GGACTACCAGGGTATCTAATCCTGTT-3′.

The thermal profile used for the reaction included a heat activation of the enzyme at 95°C for 2 min, followed by 40 cycles of denaturation at 95°C for 15 s and annealing/extension at 60°C for 60 s, followed by melt analysis ramping at 60–95°C. All measurements were taken in the log phase of amplification. Standard curves obtained using a 10-fold dilution series of bacterial DNA standards (Femto bacterial DNA quantification kit, Zymo research) ranging from 2 ng to 200 fg were routinely run with each sample set and compared with previous standard curves to check for consistency between runs. Amplicon quality was ascertained by melting curves. Amplifications of samples and standard dilutions were performed in triplicate on the StepOne Real-Time PCR System (Applied Biosystems by Life Technologies). Bacterial DNA levels were expressed as ng per ml of whole blood.

A series of controls both *in silico* and *in vitro* was performed to exclude artifacts from sample manipulation, reagent contamination and non–specific amplifications. The primers were checked for possible cross–hybridization with genes from eukaryotic and mitochondrial genomes using the database similarity search program BLAST. The BLAST search results showed no hits thus confirming the specificity of primers for the bacterial 16S rRNA as previously reported (Nadkarni et al., 2002). Separate working areas for real–time PCR mix preparation, template addition, and performing the PCR reactions were used and all experimental procedures were performed under a laminar flow hood by using dedicated pipettes, filter–sealed tips and plasticware DNA–free guaranteed. In addition, samples were processed in random order and replicated by using different reagent batches. Negative controls, in which ultrapure water instead of DNA was added, were also run in each plate. Compared with bacterial DNA detected in the blood, the levels of negative template controls were missing or very low (Supplementary Figure 1). In particular, when amplification of these controls resulted in a value about 0.05 pg, the run was discarded and the samples re–analyzed, meanwhile when values were less than 0.05 pg, they were subtracted as background from all the analyzed samples.

### Statistical Analysis

To assure the MARK–AGE data quality the following cleaning strategy was performed (1) clearing of missing values, (2) removal of outliers and (3) detection of batch effects. If detected, any batch effect for each biomarker was corrected (Baur et al., 2015).

Continuous variables were described using means and SD or SEM. Due to positively skewed values, several variables (bacterial DNA levels, whole blood leukocytes, neutrophils, lymphocytes, monocytes and platelet counts, red blood cells, C–Reactive Protein (CRP), homocysteine, fibrinogen hemoglobin, ferritin, iron and transferrin serum levels, insulin, glucose, creatinine, FFA, total cholesterol, High–Density Lipoprotein (HDL), Low–Density Lipoprotein (LDL), triglycerides, albumin and Body Mass Index (BMI) failed to pass the Kolmogorov–Smirnov test and Shapiro–Wilk test for the normal distribution. ANOVA (after correction for confounding factors) was used to evaluate differences in bacterial DNA levels among age classes (35–44; 45–54; 55–64; 65–75 years), countries and gender or differences in FFA, glucose, insulin levels, and leukocyte counts among bacterial DNA quartiles. Linear regression analysis using a stepwise method was carried out to explore the main predictors of bacterial DNA levels. The variables inserted were: circulating bacterial DNA, age, gender, country, BMI, whole blood leukocytes, neutrophil, lymphocyte, monocyte and platelet counts, red blood cells, systemic inflammation parameters (CRP, homocysteine, fibrinogen), hemoglobin (Hb) levels, ferritin, iron and transferrin serum levels, insulin, glucose, creatinine, FFA, total cholesterol, HDL, LDL, triglycerides and albumin.

All the analyses were performed using the SPSS/Win program (SPSS Statistics V22.0, Chicago, IL).

## Results

### Characteristics of Participants and Identification of Bacterial DNA in Blood RASIG Subjects From Different European Regions

The main characteristics of the study population, subdivided by gender, were reported in Supplementary Table 1. 88% of females and 90.3% of males declared a good or very good/excellent health status.

The abundance of blood microbiome in 1285 RASIG subjects from several different geographical regions of Europe was determined by quantification of 16S rRNA gene copies through quantitative real-time PCR reactions. Negative control samples, routinely run with each sample set, confirmed the lack of contamination of each step of the experimental procedure, since their detected fluorescence signal was very low compared with that of blood samples or even absent (see section “Materials and Methods”). The performance of each qPCR assay was carefully assessed so that only runs in which calibration curved displayed a correlation coefficient (R^2^) of 0.99, a mean slope of about 3.3 and a PCR efficiency > 95% with no significant intra-plate variation, were accepted. The distribution of bacterial DNA levels concerning gender and geographical origin was broad, with Germany and Poland showing significantly higher values than Belgium and Austria (*p* < 0.05), while other countries, such as Italy, Greece and Finland showed intermediate levels (Table 1). The main differences among countries were observed in male subjects.

**TABLE 1:** Bacterial DNA levels in sex stratified healthy human subjects from different European countries.

Countries	Gender	Mean	Standard error	N
Germany	Females	180.25^§^	23.66	138
	Males	143.21	20.49	114
	Total	163.49**^/§^	15.94	252
Belgium	Females	82.14	10.61	68
	Males	78.08	10.82	68
	Total	80.11	7.55	136
Poland	Females	158.28	21.47	88
	Males	111.96*	16.82	99
	Total	133.76*	13.54	187
Greece	Females	108.81	13.33	117
	Males	149.76**	18.27	102
	Total	127.88*	11.16	219
Austria	Females	88.763	8.28	100
	Males	81.30	11.18	97
	Total	85.08	6.92	197
Italy	Females	121.27	15.26	113
	Males	114.67*	13.93	120
	Total	117.87*	10.29	233
Finland	Females	102.72	12.79	42
	Males	139.99*	38.78	19
	Total	114.33*^/§^	14.91	61

^§^ p < 0.01 vs. Belgium. *p < 0.05 vs. Austria. **p < 0.01 vs. Austria. ANCOVA analysis corrected for age.

**FIGURE 1:**
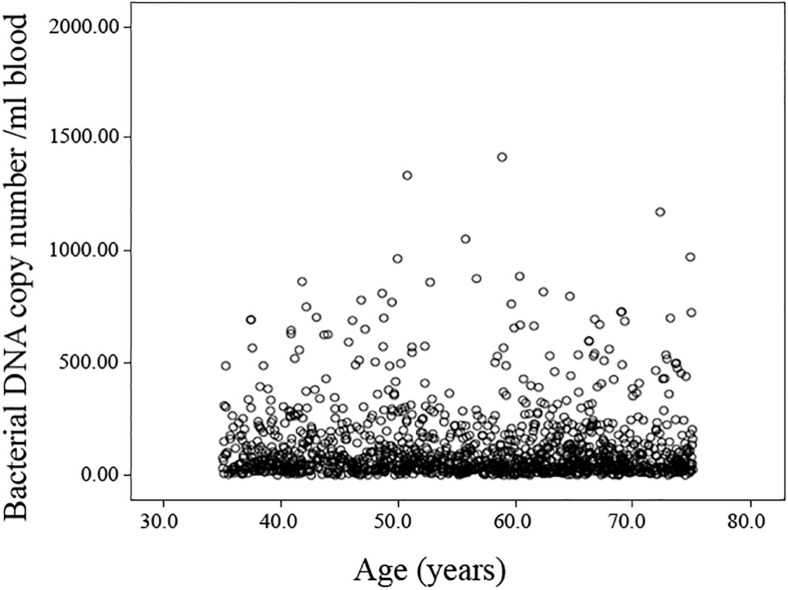
Scatterplot of the bacterial DNA levels against the age of the analyzed population. Linear regression between bacterial DNA levels (log-transformed data) and age after adjusting for gender, country and BMI, smoke habits and Self-rated health status shows no significant association (Standardized Beta coefficient = –0.039, *p* = 0.264).

The presence of bacterial DNA was detected in all samples analyzed (121.64 ± 4.85 mean and standard error), but no significant association between 16S bacterial rDNA abundance and age (Figure 1 and Supplementary Table 2) or gender (Supplementary Table 3) emerged. Likewise, no age-related difference in the bacterial DNA amount was observed even when stratifying the sample by geographical origin (data not shown). The comparison of bacterial DNA abundance was also carried out in relation to SRH, but no difference was observed.

### Bacterial DNA Levels and Hematological and Clinical-Chemical Parameters

Linear regression analysis with a stepwise approach was run in the population sample including bacterial DNA levels as the dependent variable and as independent variables several demographic, biochemical and laboratory parameters (see section “Statistical Analysis”). A significant positive association was found with leukocyte count (Standardized Beta coefficient = 0.110, *p* < 0.01) and FFA levels (Standardized Beta coefficient = 0.076, *p* < 0.05) (Table 2).

**TABLE 2:** Multivariate stepwise linear regression analysis for variables independently associated with bacterial DNA levels in the population sample.

	Unstandardized coefficients		Standardized coefficients	*P*-value
	B	Standard error	Beta	
Leukocyte count	0.298	0.099	0.110	0.003
FFA	0.206	0.080	0.076	0.010

### Blood Parameters in Relation to Quartiles of Bacterial DNA Levels

**FIGURE 2:**
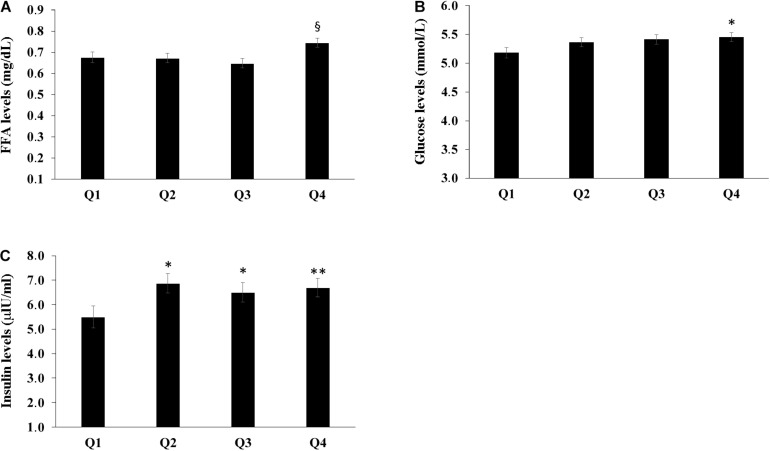
Relationship between bacterial DNA quartiles and **(A)** FFA levels, **(B)** glucose levels, and **(C)** insulin levels. The fourth quartile (Q4) of bacterial DNA levels showed the highest levels of FFA as compared with the other quartiles. The first quartile had lower insulin levels than the other quartiles and decreased glucose levels when compared with the fourth quartile. ANCOVA analysis correcting for age, countries, gender, BMI, smoke habits and Self-rated health status was applied; data are reported from the model adjusted mean ± Standard Error of the Mean (SEM); *p < 0.05 as compared to Q1; **p < 0.01 as compared to Q1; ^§^*p* < 0.05 as compared to Q1, Q2, Q3.

**FIGURE 3:**
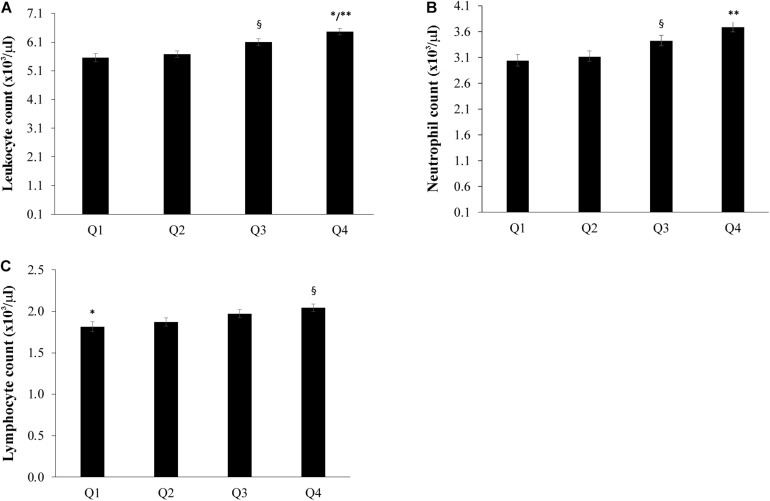
Relationship between bacterial DNA quartiles and **(A)** whole blood leukocytes, **(B)** neutrophil count, and **(C)** lymphocyte count. The fourth quartile (Q4) of bacterial DNA levels displayed the highest levels of leukocytes, lymphocytes and neutrophils than the other quartiles. ANCOVA analysis correcting for age, countries, gender, BMI, smoke habits and Self-rated health status was applied; data are reported from the model adjusted mean ± Standard Error of the Mean (SEM); ^∗∗^*p* < 0.001 as compared to Q1 and Q2; ^§^*p* < 0.05 as compared to Q1 and Q2; ^∗^*p* < 0.05 as compared to Q3.

The population sample was divided into four groups according to the quartiles of bacterial DNA levels, and the relationship with FFA, glucose and insulin levels was evaluated. After adjusting for age, countries, gender, BMI, smoke habits and Self-rated health status we found that the group in the fourth quartile (Q4) appeared to have higher levels of FFA when compared with the first and the second quartiles (*P* < 0.05) (Figure 2A). Furthermore, subjects in the first quartile showed lower insulin levels for the fourth quartile (*P* < 0.05) (Figure 2B) and decreased glucose levels when compared with the fourth quartile (*P* < 0.05) (Figure 2C). The number of total leukocytes, lymphocytes and neutrophils according to the quartiles of bacterial DNA levels was also analyzed. The fourth quartile (Q4) showed increased leukocyte, lymphocyte and neutrophil counts as compared to the lowest quartiles (Q1, Q2) (Figure 3). The same analyzes were carried out stratifying by gender and showed similar results (Supplementary Tables 4–9).

## Discussion

Several studies stated the presence of a microbial community in the blood of healthy individuals. Here, we report the results of a pilot study that, to our knowledge, represents the first quantitative description of the above population in 1285 subjects aged between 35 and 75 years recruited within the EU Project MARK-AGE with a different geographical origin, in whom a series of clinical biochemical parameters was determined. The presence of this microbiome in the blood is not surprising, since a number of reports from the literature is revealing its presence in both physiological and pathological conditions. Its origin, however, remains undefined although several data suggest a translocation into the circulatory system from different body sites, namely the gastrointestinal tract, skin, oral cavity, or translocation driven by cells of the immune system.

Our study allows assessing inter-individual variations in the extent of blood microbiome significantly associated with geographical origin rather than the age of the subject analyzed. This observation, together with the genetic peculiarities of each geographical area, could be the reflection of cultural/behavioral features like diet, environmental exposure, climate, and social habits. This suggests that the environment has a greater contribution than chronological age, at least up to 75 years old, and biological age (data not shown) (Mobeen et al., 2018). Nonetheless, an association between blood microbiome and more advanced age cannot be excluded, since dysbiosis and intestinal malabsorption as well as dysregulation of the immune response leading to a chronic systemic inflammatory state are common features of oldest-old subjects.

In our population, we observed a positive association between bacterial DNA levels and serum FFA levels by multivariate stepwise linear regression analysis. These results are in agreement with literature data reporting that gut microbiota composition is related to various lipoprotein particles and gut microbiota dysbiosis is associated with altered lipid metabolism and an increased expression of key genes involved in FFA synthesis, transport and triglyceride synthesis (TG) in the liver (Jin et al., 2016; Vojinovic et al., 2019). Likewise, a role played by FFA as mediators between metabolic conditions and the immune system has been described (Sindhu et al., 2016; Olmo et al., 2019). All in all, we may speculate that FFA in association with blood microbiome levels may represent active mediators of some biological processes. Furthermore, we observed that circulating bacterial DNA was also positively associated with total leukocytes, and the highest quartile of bacterial DNA showed an increased number of lymphocytes and neutrophils both in the whole population and after gender stratification. This finding is consistent with the well-established role played by these specialized cells in killing invading microorganisms. In addition, the strong correlation between circulating bacterial DNA and leukocyte populations may be explained by a cross-talk between the intestinal microbiota and bone marrow during bacterial infection, which induces increased myelopoiesis (Balmer et al., 2014). Moreover, circulating lipopolysaccharide is associated with activation and proliferation of lymphocyte subsets as observed for NK cells and B-cells (Ramendra et al., 2019; Sakaguchi et al., 2020). Interestingly, high levels of blood bacterial DNA were found associated with increased insulin and glucose levels. Increased insulin, a marker for insulin resistance, has been implicated in gut permeability, and impaired fasting glucose subjects show higher markers of gut permeability than controls (Carnevale et al., 2017; Mkumbuzi et al., 2020). On the other hand, gut and oral microbiota dysbiosis has been found in type 2 diabetes (DM2) subjects and different microbiome compartmentalization has been observed in DM2 (Brown et al., 2020; Shi et al., 2020). Therefore, increased circulating DNA in healthy subjects could also represent a predictor of pre-diabetes or diabetes conditions. Since our results did show differences in bacterial DNA levels among SRH categories, future longitudinal studies would be useful to clarify if persistent high levels of bacterial DNA levels in blood may represent a risk factor to develop DM2 or other age-associated diseases.

We are confident that the study was adequately controlled to avoid DNA contamination in extraction kits and laboratory reagents, which could lead to erroneous conclusions and thus significantly influence the results. In addition, the low or absent levels of DNA detected in negative template controls make us rest assured of the reliability of the results.

Finally, we believe that this explorative study is useful in providing the groundwork in the future qualitative characterization of the blood microbiome composition and a potential correlation of the microbial diversity with different phenotypes thus providing useful knowledge for both basic and clinical research.

## Data Availability Statement

The raw data supporting the conclusions of this article will be made available by the authors, without undue reservation.

## Ethics Statement

This study was approved by European Commission (Project Full Name: European Study to Establish Biomarkers of Human Ageing; Project Acronym: MARK-AGE; Project No: 200880. The patients/participants provided their written informed consent to participate in this study.

## Author Contributions

DB and MP: conceptualization. PD’A and FP: methodology. RG, MM, and MV: software. TG and NB: biobank. BG-L, OT, FD-C, CS, JB, EG, CF, MC, ES, and AH: recruitment of subjects. PD’A and RG: validation and investigation. DB, MP, GP, AB, and MV: formal analysis. AB and DB: resources. RG, MM, PD’A, EJ, MD, and ES: data curation. DB and PD’A: writing original draft preparation. PD’A, RG, DB, MP, AB, MV, and MM: writing, review, and editing. DB, MP, and GP: supervision. AB: project administration and funding acquisition. All authors have read and agreed to the published version of the manuscript.

## Conflict of Interest

 CS and JB were employed by the company BioTeSys GmbH. The authors declare that this study received funding from the European Commission, the Italian Health Ministry and the nursing homes of SADEL S.p.A. The funder was not involved in the study design, collection, analysis, interpretation of data, the writing of this article or the decision to submit it for publication.

## Publisher’s Note

All claims expressed in this article are solely those of the authors and do not necessarily represent those of their affiliated organizations, or those of the publisher, the editors and the reviewers. Any product that may be evaluated in this article, or claim that may be made by its manufacturer, is not guaranteed or endorsed by the publisher.
