# Supplementary material for: Microbiome in Blood Samples From the General Population Recruited in the MARK-AGE Project: A Pilot Study
Source: Front Microbiol. 2021 Jul 26;12:707515. doi: 10.3389/fmicb.2021.707515 (PMC8350766; doi:10.3389/fmicb.2021.707515)
Supplement: Supplementary file 1 [file Data_Sheet_1.PDF]

SUPPLEMENTARY MATERIAL

Supplementary Figure 1. Graphical representation of 16S rRNA copy number expressed in picograms (pg) quantified in negative controls (0.028±0.0017 mean±Standard Error) and blood sample (37.17±1.15 mean±Standard Error) analyzed in the study.

Supplementary Table 1. Characteristics of subjects selected from the whole MARK-AGE population for bacterial DNA analysis in blood samples

		**Females n.782**	**Males n.503**	**p value**
Age (yrs)		56.0±0.42	56.3±0.5	NS
		60.8%	39.2%	<0.01
RBC ($x10^6$/ µl)		4.53±0.02	4.94±0.02	<0.001
Hemoglobin (g/dl)		13.47±0.04	14.93±0.06	<0.001
WBC($x10^3$/µl)		5.86±0.06	6.48±0.26	NS
Neutrophils ($x10^3$/µl)		3.28±0.06	3.66±0.14	NS
Lymphocytes($x10^3$/µl)		1.93±0.02	2.06±0.10	NS
Monocytes ($x10^3$/µl)		0.43±0.17	0.51±0.18	<0.01
Platelets ($x10^3$/µl)		252.0±2.4	217.1±2.2	<0.001
Albumin (g/dl)		4.04±0.03	4.12±0.02	<0.01
CRP (µg/L)		1.98±0.11	2.10±0.13	<0.01
TC (mmol/L)		5.48±0.09	5.69±0.08	<0.01
LDL (mmol/L)		3.28±0.03	3.34±0.04	NS
TG (mmol/L)		1.14±0.03	1.41±0.04	<0.001
FG (mmol/L)		5.06±0.03	5.37±0.05	<0.001
HbA1c (%)		6.00±0.02	5.93±0.03	<0.01
BMI		25.6±0.20	26.7±0.15	<0.001
Current smoker		13%	25%	<0.01
Self-Rated Health Status	Excellent/Very good	47.3%	54%	NS
	Good	40.7%	36.3%	
	Fair/Poor	12%	9.7%	

Data are reported as mean ± Standard Error for continuous variables (Age, BMI) and percentage for categorical variables (gender, group, BMI cat, country, Self-Rated Health Status (SRH) categories).

*p-value: one-way-ANOVA (continuous variables) and Chi-square test (categorical variables).

NS = Not Significant different

The laboratory parameter analysis was adjusted for countries.

RBC: Red Blood Cells; WBC: White Blood Cells; CRP: C-Reactive Protein; TG: Triglycerides; TC: Total Cholesterol; LD: Low-Density Lipoprotein; FG: Fasting Glucose; HbA1c: Hemoglobin A1c; BMI: Body Mass Index.

Supplementary Table 2. Bacterial DNA levels in blood samples in relation to age classes

Age Classes	Bacterial DNA levels Mean	Std. Error	95% Confidence Interval	
			Lower Bound	Upper Bound
35-44	138.290	12.428	113.909	162.672
45-54	127.916	11.185	105.974	149.858
55-64	115.314	9.939	95.816	134.812
65-75	129.332	10.349	109.030	149.635

$p > 0.05$ among age classes

ANCOVA analysis correcting for countries, gender, BMI, smoke habits and Self-Rated Health status was applied

Supplementary Table 3. Bacterial DNA in blood samples in relation to gender

Gender	Bacterial DNA levels Mean	Std. Error	95% Confidence Interval	
			Lower Bound	Upper Bound
Females	131.874	8.272	116.645	149.102
Males	121.553	8.714	105.458	139.647

$p > 0.05$ between males and females

ANCOVA analysis correcting for countries, age, BMI, smoke habits and Self-Rated Health Status was applied

Supplementary Table 4. Relationship among bacterial DNA quartiles and glucose levels

Gender	Bacterial DNA quartiles	FG (mmol/L) Mean	Std. Error	95% Confidence Interval	
				Lower Bound	Upper Bound
Females	Q1	5.078	0.085	4.910	5.245
	Q2	5.215	0.080	5.058	5.372
	Q3	5.169	0.073	5.026	5.312
	Q4	5.400*	0.076	5.150	5.550
Males	Q1	5.176	0.130	5.120	5.632
	Q2	5.512	0.130	5.256	5.768
	Q3	5.675	0.137	5.405	5.945
	Q4	5.556*	0.124	5.311	5.800

*$p < 0.05$ as compared to Q1 quartile of bacterial DNA levels

ANCOVA analysis correcting for age, countries, BMI, smoke habits and Self-Rated Health Status was applied; FG: Fasting Glucose

Supplementary Table 5. Relationship among bacterial DNA quartiles and insulin levels after gender stratification

Gender	Bacterial DNA quartiles	Insulin (µIU/ml) Mean	Std. Error	95% Confidence Interval	
				Lower Bound	Upper Bound
Females	Q1	6.019	0.516	5.005	7.033
	Q2	6.446	0.477	6.508	7.384
	Q3	5.687	0.434	4.834	6.539
	Q4	6.067	0.440	5.202	6.932
Males	Q1	5.951	0.473	5.020	6.881
	Q2	6.568	0.484	5.616	7.520
	Q3	6.962	0.502	5.976	7.948
	Q4	6.887*	0.440	6.022	7.752

*p<0.05 as compared to Q1 quartile of bacterial DNA levels

ANCOVA analysis correcting for age, countries, BMI, smoke habits and Self-Rated Health Status was applied

Supplementary Table 6. Relationship among bacterial DNA quartiles and FFA levels after gender stratification

Gender	Bacterial DNA quartiles	FFA(mg/dl) Mean	Std. Error	95% Confidence Interval	
				Lower Bound	Upper Bound
Females	Q1	0.685	0.026	0.633	0.736
	Q2	0.656	0.026	0.606	0.706
	Q3	0.727	0.024	0.680	0.774
	Q4	0.733*	0.026	0.682	0.784
Males	Q1	0.666	0.029	0.610	0.722
	Q2	0.688	0.029	0.632	0.744
	Q3	0.633	0.030	0.575	0.691
	Q4	0.719°	0.029	0.663	0.775

*p<0.05 as compared to Q1, Q2 quartiles of bacterial DNA levels

°p<0.05 as compared to Q1, Q3 quartiles of bacterial DNA levels

ANCOVA analysis correcting for age, countries, BMI, smoke habits and Self-Rated Health Status was applied

Supplementary Table 7. Relationship among bacterial DNA quartiles and whole blood leukocytes after gender stratification

Gender	Bacterial DNA quartiles	WBL ($x10^3$/µl) Mean	Std. Error	95% Confidence Interval	
				Lower Bound	Upper Bound
Females	Q1	5.562	0.167	5.234	5.891
	Q2	5.816	0.156	5.509	6.124
	Q3	6.114*	0.140	5.840	6.388
	Q4	6.387**	0.142	6.106	6.667
Males	Q1	6.327	0.170	5.992	6.662
	Q2	6.225	0.173	5.884	6.566
	Q3	6.406	0.178	6.056	6.756
	Q4	6.934°	0.155	6.629	7.239

*p<0.01 as compared to Q1 quartile of bacterial DNA levels

**p<0.01 as compared to Q1, Q2 quartiles of bacterial DNA levels

°p<0.01 as compared to Q1, Q2, Q3 quartiles of bacterial DNA levels

ANCOVA analysis correcting for age, countries, BMI, smoke habits and Self-Rated Health Status was applied; WBL: Whole Blood Leukocytes

Supplementary Table 8. Relationship among bacterial DNA quartiles and neutrophil count after gender stratification

Gender	Bacterial DNA quartiles	Neutrophils ($x10^3$/µl) Mean	Std. Error	95% Confidence Interval	
				Lower Bound	Upper Bound
Females	Q1	2.945	0.165	2.620	3.269
	Q2	3.075	0.155	2.772	3.379
	Q3	3.503§	0.138	3.232	3.775
	Q4	3.570*	0.141	3.293	3.846
Males	Q1	3.578	0.126	3.330	3.825
	Q2	3.518	0.128	3.266	3.769
	Q3	3.587°	0.132	3.327	3.846
	Q4	3.964**	0.114	3.740	4.189

*p<0.05 as compared to Q1 quartile of bacterial DNA levels

**p<0.01 as compared to Q1, Q2, Q3 quartiles of bacterial DNA levels

°p<0.01 as compared to Q1 quartile of bacterial DNA levels

§P<0.05 as compared to Q1, Q2 quartiles of bacterial DNA levels

ANCOVA analysis correcting for age, countries, BMI, smoke habits and Self-Rated Health Status was applied

Supplementary Table 9. Relationship among bacterial DNA quartiles and lymphocyte count after gender stratification

GENDER	Bacterial DNA quartiles	Lymphocytes ($x10^3/\mu l$) Mean	Std. Error	95% Confidence Interval	
				Lower Bound	Upper Bound
Females	Q1	1.900	0.061	1.780	2.021
	Q2	2.011	0.057	1.898	2.124
	Q3	1.988	0.052	1.887	2.089
	Q4	2.094*	0.053	1.991	2.198
Males	Q1	1.938	0.080	1.780	2.095
	Q2	1.959	0.081	1.799	2.120
	Q3	2.044	0.084	1.879	2.210
	Q4	2.122*	0.073	1.979	2.265

*$p<0.05$ as compared to Q1 quartile of bacterial DNA levels

ANCOVA analysis correcting for age, countries, BMI, smoke habits and Self-rated health status was applied

Supplementary Table 10. Bacterial DNA levels in blood samples from RASIG subjects in relation to the Self-Rated Health Status (SRH) and after gender stratification

Gender	SRH	Bacterial DNA levels Mean	Std. Error	95% Confidence Interval	
				Lower Bound	Upper Bound
Females	Excellent/ Very good	119.133	8.676	102.097	136.169
	Good	124.586	8.971	106.970	142.201
	Fair/Poor	124.840	15.391	94.616	155.063
Males	Excellent/ Very good	127.940	7.273	113.657	142.223
	Good	136.777	8.528	120.030	153.525
	Fair/Poor	109.087	15.397	78.848	139.326

$p>0.05$ among SRH categories

ANCOVA analysis correcting for age, countries, BMI, smoke habits and Self-Rated Health Status was applied
