# Supplementary figures and images for: Microbiome in Blood Samples From the General Population Recruited in the MARK-AGE Project: A Pilot Study
Source: Front Microbiol. 2021 Jul 26;12:707515. doi: 10.3389/fmicb.2021.707515 (PMC8350766; doi:10.3389/fmicb.2021.707515)

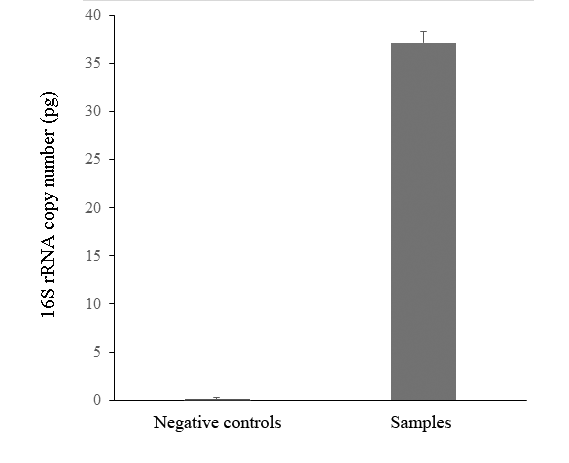

Supplement: Supplementary file 2 [file Image_1.TIF]
